# Domain-Specific DNA Binding Activities of BRCA1 Reveal Substrate Preferences for Homologous Recombination and Telomere Regulation

**DOI:** 10.1021/acs.biochem.5c00333

**Published:** 2025-09-04

**Authors:** Kaitlin Lowran, Laura Campbell, Emma Cismas, Colin G. Wu

**Affiliations:** Department of Chemistry, 6918 Oakland University, Rochester, Michigan 48309, United States

## Abstract

BRCA1 is a crucial component of homologous recombination (HR), a high-fidelity pathway for repairing double-stranded DNA breaks (DSBs) in human cells. The central region of the BRCA1 protein contains two putative DNA binding domains (DBDs), yet their relative substrate specificities and functional contributions to HR remain unclear. Here, we characterized the DNA binding properties of DBD1 (amino acids 330–554), DBD2 (amino acids 894–1057), and BRCA1 C-terminal (BRCT) repeats using biolayer interferometry. Affinities were determined for single-stranded DNA (ssDNA), double-stranded DNA (dsDNA), and G-quadruplex (G4) DNA. DBD2 exhibited strong and nearly identical binding to all three substrates (*K*_d_ = ∼35–44 nM), while the BRCT also bound to each structure similarly, but with lower affinity (*K*_d_ = ∼149–184 nM). In contrast, DBD1 showed a distinct preference for dsDNA, binding approximately 2-fold tighter compared to ssDNA or G4. These findings support a model in which BRCA1 uses modular DNA binding domains to recognize diverse repair targets; DBD2 serves as a primary anchor to associate with a broad range of DNA structures with BRCT contributing to the contacts. DBD1 acts as the determinant of DNA structure-specific localization that may help direct BRCA1 to DSB sites during HR or to noncanonical elements such as chromatin and telomeres. These insights lay the groundwork for future studies examining how cancer-associated variants affect the DNA binding and repair phenotypes of BRCA1 and may inform the interpretation of variants of unknown clinical significance.

## Graphical abstract

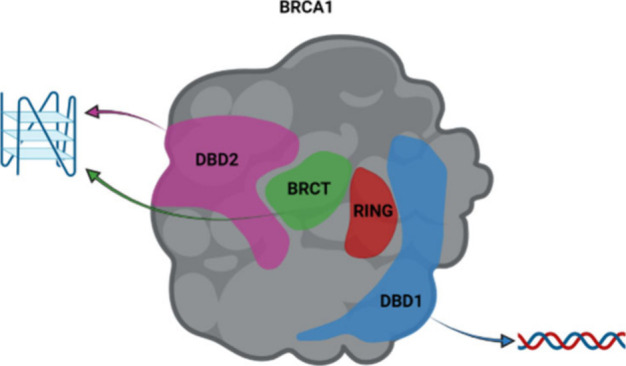

## Introduction

The Breast Cancer Susceptibility Gene 1 (*BRCA1*) encodes a large tumor suppressor protein that is essential for maintaining genomic integrity. BRCA1 plays pivotal roles in the repair of double-stranded DNA breaks (DSB), transcriptional regulation, and telomere maintenance.^1–4^ Germline mutations in *BRCA1* are associated with elevated risks of breast and ovarian cancers as well as cardiovascular diseases.^5,6^ BRCA1 facilitates DSB repair during homologous recombination (HR) by coordinating the recruitment of DNA end-resection proteins such as 53BP1.^3,7,8^ This is subsequently followed by interaction with PALB2 to promote BRCA2/RAD51-mediated strand invasion.^9–11^ Additionally, BRCA1 stabilizes stalled replication forks and promotes fork recovery during replication stress.^12,13^

Replication stress arises from obstacles such as DNA damage, common fragile sites, and noncanonical DNA structures.^14^ If left unresolved, these impediments can lead to fork collapse and the formation of DSB, which must be repaired timely to prevent genome instability and tumorigenesis.^15–17^ While there are multiple DSB repair pathways—including nonhomologous end joining (NHEJ), alternative end joining, and single-strand annealing—HR is the predominant mechanism and is favored for its high fidelity.^18–21^

Noncanonical DNA structures, such as D-loops, cruciform, G-quadruplexes (G4), and other fork intermediates, can arise during replication and transcription and they must be resolved.^22^ BRCA1 binds to all of these structures,^23–26^ a function that may be relevant to its roles in telomere regulation and satellite DNA maintenance.^2,4,27^ Notably, BRCA1-deficient cells are hypersensitive to G4-stabilizing compounds such as CX-5461, which impedes replication fork progression and induces DNA breaks.^28,29^

The central region of BRCA1 is intrinsically disordered, and it is flanked by structured domains at the N- and C-termini that are well-folded. The N-terminal RING domain forms a heterodimer with the BARD1 tumor suppressor and functions as an E3 ubiquitin ligase.^30–32^ The BRCA1–BARD1 complex binds preferentially to D-loops and DNA bubbles, facilitating RAD51-mediated HR and replication fork restart at G4 regions.^24,33^ In contrast, the C-terminal BRCT is a phosphoprotein recognition site and is prevalent in other DNA repair proteins.^34^ BRCA1 binds to the FANCJ DNA helicase via BRCT when FANCJ is phosphorylated on serine 990. Formation of the BRCA1–FANCJ complex may be needed for the conversion of interstrand cross-links (ICL) to DSB for repair by homologous recombination and to promote replication restart at G4 sites.^35–38^ Beyond these roles, the BRCT domain also interacts with supercoiled DNA, contributes to chromatin remodeling, and participates in the detection of DNA breaks.^39–42^ It suppresses ribosomal R-loops by facilitating sense–antisense rRNA pairing and enhancing RNA polymerase I-driven antisense rRNA transcription.^43^

The central portion of BRCA1, though unstructured, serves as a scaffold for protein and DNA interactions.^23,26,42,44^ Within this region, two discrete DNA binding domains (DBD) have been identified: DBD1 (amino acids 330–554) and DBD2 (amino acids 894–1057).^40,43,45–47^

Although the relative affinities of the DBD for branched DNA substrates have been examined, the results were mostly qualitative due to the presence of multiple BRCA1–DNA complexes, which made quantitative estimates of the affinities difficult.^23^ To further characterize the DNA binding properties of these domains, we measured the affinities of DBD1, DBD2, and BRCT for three DNA substrates that are representative of repair intermediates: single-stranded DNA (ssDNA), double-stranded DNA (dsDNA), and a human telomeric G4. Our findings suggest that the BRCA1 DBD are functionally specialized, with distinct substrate specificities that may direct BRCA1 protein to either DSB or to the telomere during the DNA damage response.

## Materials and Methods

### Buffers and Reagents

All solutions were prepared with analytical-grade chemicals and Type I ultrapure water from a Smart2Pure 6 UV/UF system (ThermoFisher; Waltham, MA, USA). The solutions were then sterilized through a 0.22 μm PES filter prior to use.

### Expression and Purification of Recombinant Proteins

Codon-optimized *E. coli* expression plasmids encoding human BRCA1 DBD1 (aa 330–554), DBD2 (aa 894–1057), and BRCT (aa 1646–1863) were purchased from VectorBuilder (Chicago, IL, USA) and validated by Sanger sequencing. Constructs were transformed into NiCo21­(DE3) competent cells (New England Biolabs; Ipswich, MA, USA). Starter cultures (125 mL) were expanded into 4 L of LB medium (1:20 dilution) that was maintained at 37 °C and 250 rpm by using a MaxQ 4000 orbital shaker (ThermoFisher). Protein expression was induced with 1 mM IPTG when the OD_600_ reached 0.6. After 4 h of expression, the cells were harvested by centrifugation using a J6-MI high-capacity centrifuge equipped with a JS-4.2 rotor (4,000 rpm at 4 °C for 90 min).

Cell pellets were resuspended in lysis buffer (20 mM NaPi (pH 7.5), 300 mM NaCl, 30 mM imidazole, 1 mM DTT, 5% (v/v) glycerol, 1% NP-40, and 1 mM PMSF), and lysed by sonication using a Fisherbrand 505 Dismembrator (Fisher Scientific; Hampton, NH, USA). Samples were chilled on ice during sonication (15 s on, 45 s off, and 45% amplitude for 15 min). Lysates were clarified by centrifugation with a Sorvall RC5C Plus high-speed centrifuge and a SS-34 fixed angle rotor (18,500 rpm at 4 °C for 90 min). The supernatant was filtered through a 0.45 μm PES membrane prior to liquid chromatography.

All protein purification steps were performed at 4 °C. Clarified lysates were incubated for 45 min with 20 mL of Ni-NTA agarose resin (Goldbio; St. Louis, MO, USA) that was pre-equilibrated in binding buffer (20 mM NaPi (pH 7.5), 300 mM NaCl, 30 mM imidazole, 1 mM DTT, and 5% glycerol). After batch binding, the resin was washed sequentially with 10 column volumes (CV) of equilibration buffer and 10 CV of low-salt buffer (20 mM NaPi (pH 7.5), 30 mM NaCl, 30 mM imidazole, 1 mM DTT, and 5% glycerol). Bound proteins were eluted with 1 CV of elution buffer (20 mM NaPi (pH 7.5), 30 mM NaCl, 300 mM imidazole, 1 mM DTT, and 5% glycerol).

Eluates were immediately loaded onto a 5 mL HiTrap Heparin HP column (GE Healthcare, Chicago, IL, USA) that was equilibrated with 20 mM HEPES (pH 7.5), 30 mM NaCl, 5 mM TCEP, and 5% glycerol using an AKTA Start FPLC system (GE Healthcare). The column was washed with 10 CV of the equilibration buffer, and the protein was eluted with 20 mM HEPES (pH 7.5), 1 M NaCl, 5 mM TCEP, and 5% glycerol over a 50 mL linear gradient in 2 mL fractions. Elution fractions were analyzed by SDS-PAGE, pooled, and dialyzed into storage buffer (20 mM HEPES pH 7.5, 150 mM KCl, 5 mM TCEP, and 20% glycerol) by using 3500 MWCO tubing (ThermoFisher). After three buffer exchanges, proteins were flash-frozen in liquid nitrogen and stored at −80 °C. Protein concentrations were measured with a NanoDrop One C spectrophotometer (ThermoFisher) using the molar extinction coefficients listed in Table 1.

**Table 1:** Amino Acid Sequences of BRCA1 DBD1, DBD2, and BRCT

name	sequence (N→C)	molar extinction coefficient (M^–1^ cm^–1^)
6xHis-BRCT (aa 1646–1861)	HHHHHHENLYFQGVNKR​MSM​VVS​GLT​PEE​FML​VYKF​ARK​HHI​TLT​NLI​TEE​TTH​VVMK​TDA​EFVC​ERT​LKYF​LGIA​GGK​WVV​SYF​WVT​QSIK​ERKM​LNE​HDF​EVRG​DVV​NGR​NHQ​GPK​RAR​ESQD​RKI​FRGL​EICC​YG​PFT​NMP​TDQ​LEWMV​QLC​GAS​VVK​ELS​SFTL​GTG​VHP​IVVV​QPD​AWT​EDNG​FHA​IGQ​MCE​APV​VTR​EWV​LDS​VAL​YQC​QEL​DTY​LIP​QIP​HS	39,420
predicted MW: 26 kDa		
pI = 6.3		
6xHis-DBD1 (aa 330–554)	HHHHHHENLYFQGDRRTP​STE​KKVD​LN​ADPL​CER​KEWN​KQK​LPC​SEN​PRD​TED​VPWI​TLN​SSI​QKV​NEW​FSRS​DELL​GSD​DSH​DGE​SES​NAK​VAD​VLD​VLN​EVD​EYS​GSS​EKID​LLA​SDP​HEA​LIC​KSE​RVHS​KSV​ESN​IED​KIF​GKT​YRK​KAS​LPN​LSH​VTE​NLI​IGA​FVT​EPQ​IIQ​ERPL​TNK​LKR​KRR​PTS​GLH​PED​FIKK​ADL​AVQ​KTP​EMI​NQG​TNQ​TEQ​NGQ​VMN​ITN​SGHE	20,970
predicted MW: 27 kDa		
pI = 5.7		
6xHis-DBD2 (aa 894–1057)	HHHHHHENLYFQGKQS​PKV​TFE​CEQ​KEE​NQG​KNE​SNI​KPV​QTV​NIT​AGF​PVV​GQK​DKPV​DNA​KCS​IKG​GSR​FCL​SSQ​FRG​NET​GLI​TPN​KHG​LLQN​PYR​IPP​LFP​ISFV​KTK​CKK​NLLE​EFE​EHS​MSP​ERE​MGN​ENI​PST​VST​ISR​NNI​REN​VFK​EAS​SSN​INE​VGS​STN​EVG​SS	2980
predicted MW: 20 kDa		
pI = 8.4		

### DNA Oligos

All oligonucleotides were synthesized by Integrated DNA Technologies (IDT; Coralville, IA, USA). Oligos were dissolved in Buffer H (20 mM HEPES pH 7.5, 150 mM KCl, 5 mM TCEP, and 5% glycerol), and DNA concentrations were determined spectrophotometrically. Double-stranded DNA was prepared by mixing the nonbiotinylated complementary strand in 1.05x molar excess over the biotinylated oligo, incubating the sample at 95 °C for 10 min, and cooling it slowly to room temperature over 6 h. A Cy3 label was included in the complementary strand to confirm the successful annealing by gel electrophoresis.

### Biolayer Interferometry (BLI)

BLI assays were performed on a BLItz instrument (Sartorius) in Buffer H at 25 °C. High precision streptavidin-coated biosensors (SAX) were purchased from Sartorius and hydrated in Buffer H for 10 min prior to use. For each experiment, the sensor was placed in Buffer H for 30 s to collect an initial baseline. Next, 150 nM of biotinylated DNA was loaded onto the sensor over 120 s and a second baseline was collected in Buffer H for 30 s. Protein samples were then introduced at different concentrations. The association reactions were monitored for 180 s, and dissociation of the complexes were examined for 120 s in Buffer H. A reference curve was obtained in the buffer alone and was subtracted from the other traces. BLI measurements were performed in triplicate on separate days. For further methodological details, see reference 48.

### Data Analysis

BLI binding isotherms were constructed by plotting the steady-state response (Δλ) values against the protein concentration. The curves were fit to a 1:1 binding model in Scientist 3.0 software (Micromath; St. Louis, MO) using the following implicit formulas (eqs 1–3), where Δλ was the observed signal change, *A* was the amplitude, and *K* was the equilibrium association constant. *D*_f_ and *D*_t_ were the free and total concentration of DNA, while *P*_f_ and *P*_t_ described the free and total concentration of the protein. The binding constants reported were determined from the average and 95% confidence intervals of three independent data sets.

1 $$\Delta \lambda =A\left(\frac{KP_{f}}{1+KP_{f}}\right)$$

2 $$D_{t}=D_{f}\left(1+KP_{f}\right)$$

3 $$P_{t}=P_{f}\left(1+KD_{f}\right)$$

## Results

### Expression and Purification of BRCA1 DNA Binding Domains

Recombinant BRCA1 DBD1 (amino acids 330–554), DBD2 (amino acids 894–1057), and BRCT domain (amino acids 1646–1861) were expressed in *E. coli* and purified to near homogeneity using tandem Ni-NTA and heparin affinity chromatography. The three proteins migrated as single bands as determined by SDS-PAGE (Figure 1). The BRCT appeared above the 25 kDa marker, consistent with a predicted molecular weight of 26 kDa. DBD1 and DBD2 both showed slightly larger apparent sizes, with DBD1 migrating as a 30–35 kDa band and DBD2 appearing between the 20–25 kDa markers. A small discrepancy in apparent molecular size was expected for intrinsically disordered proteins such as DBD1 and DBD2 due to the limitations of SDS-PAGE, but as an extra precaution, the amino acid sequence of purified DBD1 was confirmed by mass spectrometry (Supporting Figure 1).

**Figure 1:**
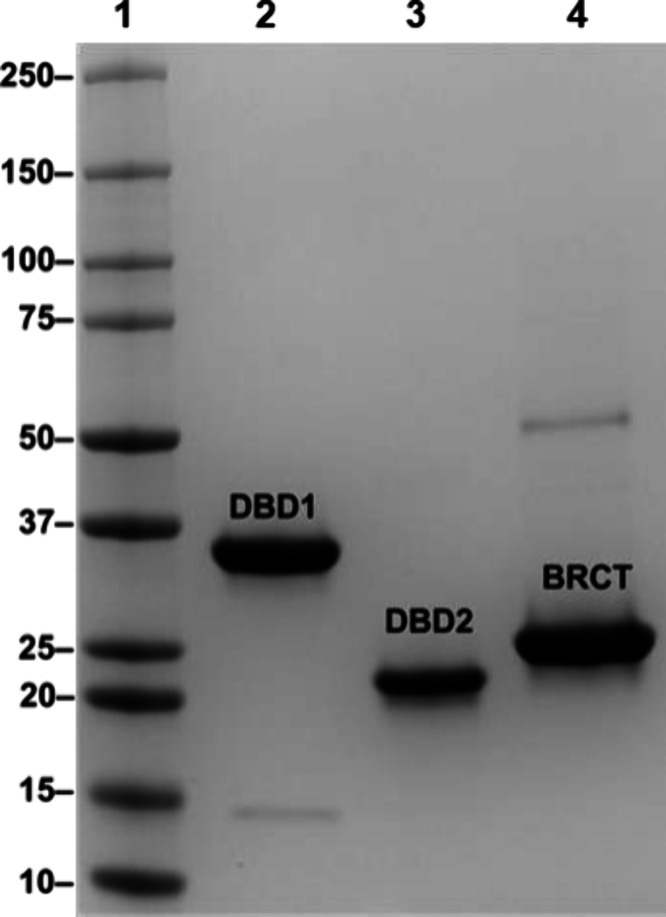
SDS-PAGE of purified recombinant BRCA1 DBD1, DBD2, and BRCT. A ladder with known molecular weight standards (in kilocalories) was loaded into Lane 1. Purified DBD1 (lane 2), DBD2 (lane 3), and BRCT (lane 4) were loaded in the respective lanes.

### Design of DNA Substrates

To examine the DNA binding properties of the BRCA1 domains, we designed three biotinylated DNA substrates, as shown in Table 2: a 60 nt ssDNA, a fully annealed blunt-end dsDNA, and a G4 formed from the human telomeric repeat sequence (TTAGGG)_4_. The ssDNA and dsDNA structures are common DSB repair intermediates during homologous recombination, while the G4 was examined due to BRCA1’s emerging role in telomere maintenance.^28,29^ Branched DNA substrates would introduce additional potential binding sites and were excluded to maintain 1:1 stoichiometry for quantitative analysis of the affinity values.

**Table 2:** DNA Oligo Sequences

name	sequence (5′→3′)	molar extinction coefficient (M^–1^ cm^–1^)	molecular weight (g/mol)
Bioss60R	/5Biosg/CCATGGCTCCTGAGCTAGCTGCAGTAGCCTAAAGGATGAAACTAGGATCTTATGCTCCAT	574,200	18,459
BioG4Telo	/5Biosg/TTAGGGTTAGGGTTAGGGTTAGGG	244,600	7575
ss60RC	/5Cy3/ATGGAGCATAAGATCCTAGTTTCATCCTTTAGGCTACTGCAGCTAGCTCAGGAGCCATGG	582,700	18,996
Biods60R	Bioss60R + ss60RC		37,849

### Biolayer Interferometry Assays

Biolayer interferometry (BLI) was used to measure the binding affinities of DBD1, DBD2, and BRCT for each DNA substrate. Figure 2A outlines a standard BLI workflow. The streptavidin-coated BLI sensors (SAX) were hydrated in Buffer H, and an initial baseline signal was measured (Phase 1). The biotinylated DNA was loaded onto the SAX biosensors, producing an increase in the binding signal (Δλ) (Phase 2). After the DNA was loaded, the sensor tip was washed in Buffer H to establish a new baseline (Phase 3). This step ensured that the DNA substrate remains bound to the SAX sensors. The proteins were then added at varying concentrations to initiate association reactions (Phase 4). After a steady-state plateau was reached, the sensor was placed into Buffer H to monitor the dissociation reactions (Phase 5). Figure 2B illustrates the association and dissociation time-courses collected from multiple protein concentrations. The plateau values were plotted against protein concentration and fit to a 1:1 interaction model using nonlinear least-squares analysis to determine the equilibrium constant (*K*) values (Figure 2C).

**Figure 2:**
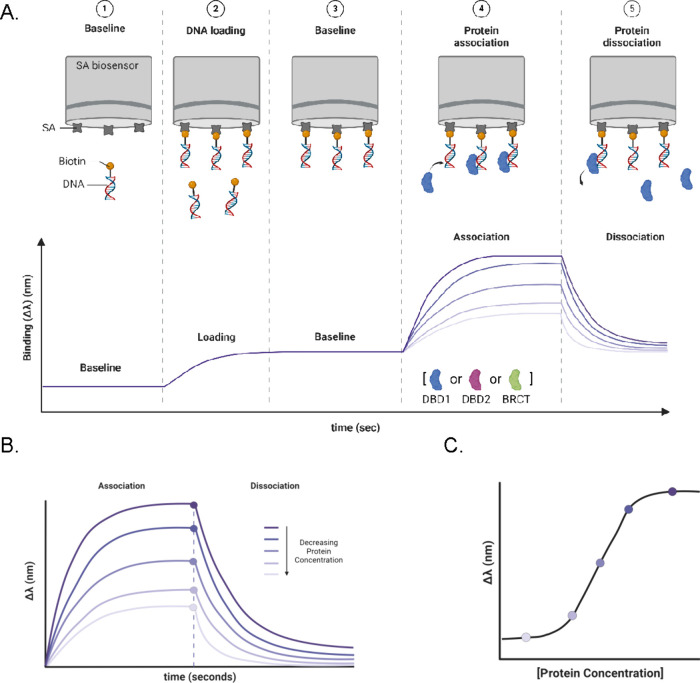
Biolayer interferometry (BLI) binding assays. (A) Cartoon depicting a time-course of each step of a BLI experiment. The interference pattern of a streptavidin-coated (SAX) biosensor was measured in Buffer H to establish a baseline (Phase 1). Biotinylated DNA was loaded onto the sensor tip through biotin–SA interactions, and an increase in Δλ was measured (Phase 2). The biosensor was washed with buffer to confirm that the DNA substrate remained surface-immobilized and to establish a new baseline (Phase 3). Protein (DBD1, blue; DBD2, magenta; BRCT, green) was introduced to initiate the association reaction (Phase 4). Once a binding equilibrium was reached, the biosensor was returned to Buffer H to examine the dissociation of the protein–DNA complexes (Phase 5). (B) shows the BLI time-courses of the association and dissociation steps as a function of protein concentration. (C) Nonlinear least-squares analysis of the steady-state BLI signals fit to a 1:1 binding model. Cartoon was prepared using BioRender.

### DNA Binding Profiles of the BRCA1 Domains

Quantitative BLI revealed distinct DNA binding patterns for each of the BRCA1 domain. DBD1 exhibited the highest binding affinity for dsDNA (*K*_A_ = 2.92 ± 0.66 × 10^7^ M^–1^; *K*_D_ = 3.55 ± 0.90 × 10^–8^ M), followed by lower affinities for ssDNA (*K*_A_ = 1.67 ± 0.65 × 10^7^; *K*_D_ = 6.84 ± 3.38 × 10^–8^ M) and for G4 DNA (*K*_A_ = 1.52 ± 0.28 × 10^7^ M^–1^; *K*_D_ = 6.72 ± 1.25 × 10^–8^ M) (Figure 3). This preference for dsDNA is consistent with the potential role of DBD1 in recognizing DSB during early HR. Although DBD1 also bound to ssDNA and to G4, these interactions were weaker compared to dsDNA by 2-fold, indicating reduced selectivity for these intermediates.

**Figure 3:**
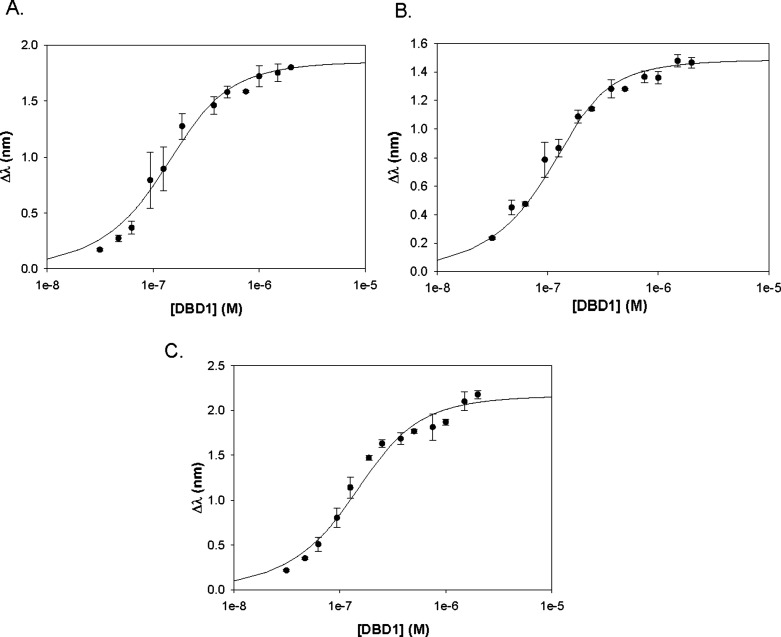
Plots of the BRCA1 DBD1. (A) ssDNA. (B) dsDNA. (C) G4 DNA.

DBD2 bound to G4 DNA (*K*_A_ = 3.26 ± 1.21 × 10^7^ M^–1^; *K*_D_ = 3.47 ± 1.61 × 10^–8^ M), ssDNA (*K*_A_ = 2.47 ± 0.23 × 10^7^ M^–1^; *K*_D_ = 4.08 ± 0.37 × 10^–8^ M), and dsDNA (*K*_A_ = 2.27 ± 0.31 × 10^7^ M^–1^; *K*_D_ = 4.46 ± 0.60 × 10^–8^ M) with similarly high affinities (Figure 4). These values were equivalent to what was observed for DBD1-dsDNA. The BRCT, described traditionally as a phosphopeptide-interaction domain, also demonstrated DNA binding activity. The equilibrium constant values of BRCT for G4 DNA (*K*_A_ = 6.82 ± 1.09 × 10^6^ M^–1^; *K*_D_ = 1.49 ± 0.26 × 10^–7^ M), ssDNA (*K*_A_ = 6.34 ± 0.49 × 10^6^ M^–1^; *K*_D_ = 1.58 ± 0.12 × 10^–7^ M), and dsDNA (*K*_A_ = 5.46 ± 0.42 × 10^6^ M^–1^; *K*_D_ = 1.84 ± 0.14 × 10^–7^ M) (Figure 5) were also nearly identical; however, they were consistently 3–5 times weaker compared to DBD2. All of the BLI binding results are summarized in Table 3.

**Figure 4:**
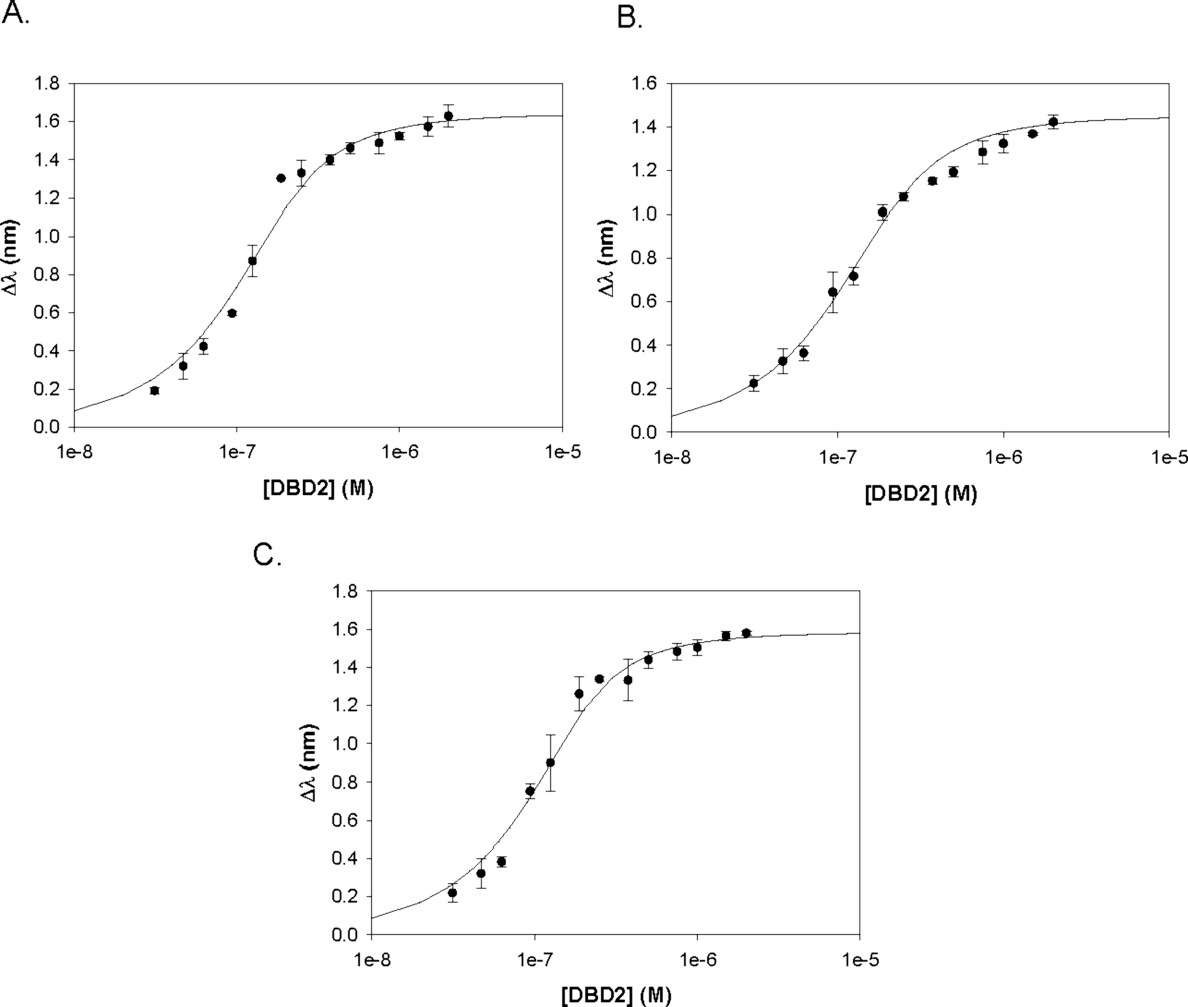
Plots of the BRCA1 DBD2. (A) ssDNA. (B) dsDNA. (C) G4 DNA.

**Figure 5:**
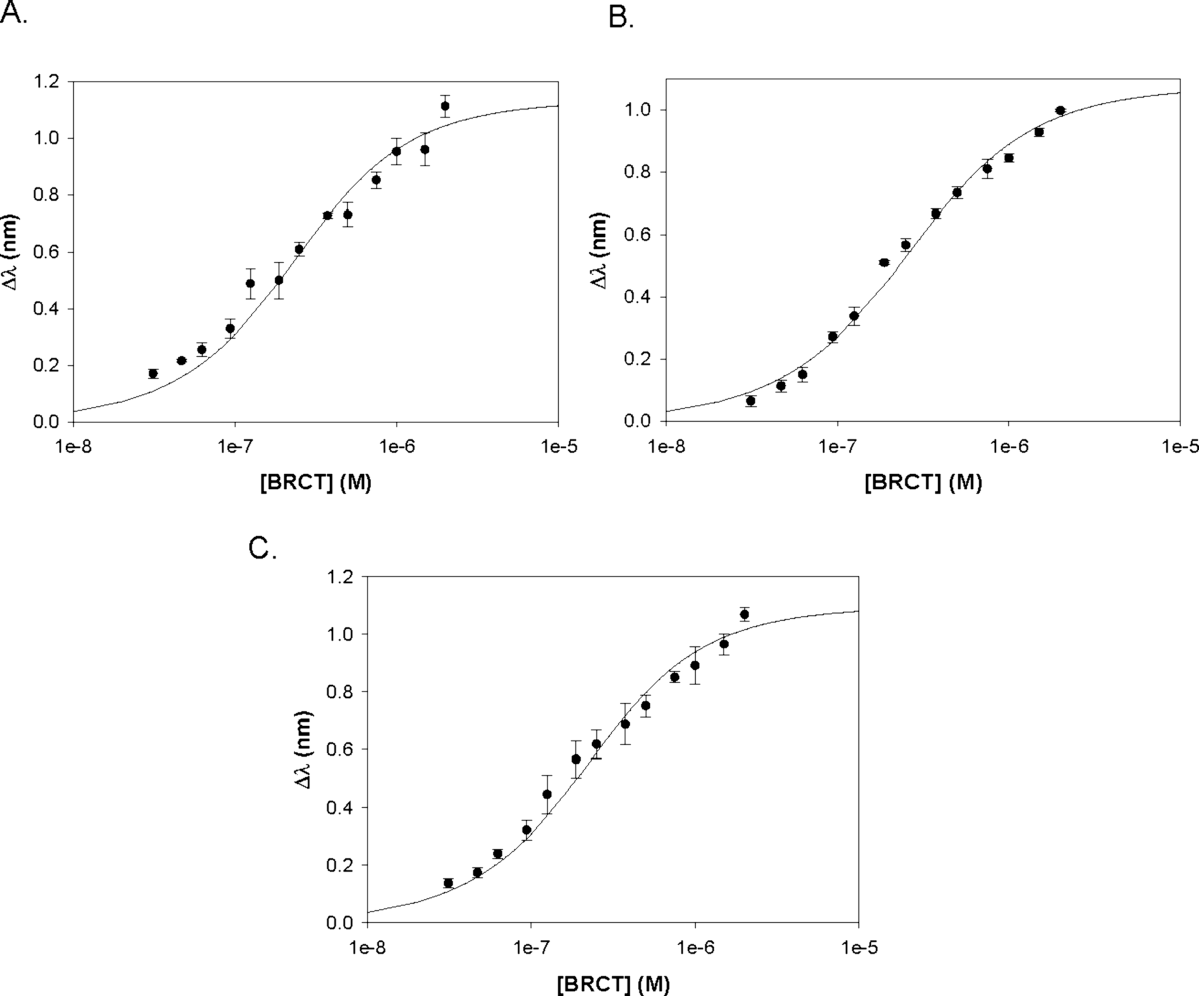
Plots of the BRCA1 BRCT. (A) ssDNA. (B) dsDNA. (C) G4 DNA.

**Table 3:** Summary of BLI Data

protein	DNA substrate	*K*_A_ (M^–1^)	*K*_D_ (M)	*A*	*R* ^2^
DBD1	ssDNA	1.67 ± 0.65 × 10^7^	6.84 ± 3.38 × 10^–8^	1.85 ± 0.037	0.995
	G4	1.52 ± 0.28 × 10^7^	6.72 ± 1.25 × 10^–8^	2.16 ± 0.044	0.995
	dsDNA	2.92 ± 0.66 × 10^7^	3.55 ± 0.90 × 10^–8^	1.48 ± 0.021	0.997
DBD2	ssDNA	2.47 ± 0.23 × 10^7^	4.08 ± 0.37 × 10^–8^	1.64 ± 0.029	0.996
	G4	3.26 ± 1.21 × 10^7^	3.47 ± 1.61 × 10^–8^	1.58 ± 0.023	0.997
	dsDNA	2.27 ± 0.31 × 10^7^	4.46 ± 0.60 × 10^–8^	1.45 ± 0.043	0.996
BRCT	ssDNA	6.34 ± 0.49 × 10^6^	1.58 ± 0.12 × 10^–7^	1.13 ± 0.027	0.993
	G4	6.82 ± 1.09 × 10^6^	1.49 ± 0.26 × 10^–7^	1.09 ± 0.021	0.995
	dsDNA	5.46 ± 0.42 × 10^6^	1.84 ± 0.14 × 10^–7^	1.07 ± 0.016	0.997

## Discussion

The central region of BRCA1 has been implicated in recognizing DNA structures, including cruciform, supercoiled, G4, and triplex DNA.^23,26,46,47^ However, the precise boundaries of the DBD have varied across the literature. Paull et al.^23^ initially defined the DBD as spanning residues 452–1079, while Brázda et al.^26^ refined it to amino acids 444–1057, and Masuda et al.^25^ described aa 421–701 as a minimal DNA binding region. Later work suggested the DBD could be subdivided—Mark et al. identified two subdomains (aa 498–663 and 936–1057), and minimal binding fragments were reported by Naseem et al. (aa 340–554) and Brázda et al. (aa 894–1057).^44,47,49^ Based on these reports, we generated BRCA1 constructs DBD1 (aa 330–554) and DBD2 (aa 894–1057) and examined their DNA binding properties alongside the BRCT (aa 1646–1861), a region primarily studied for phosphopeptide binding but also implicated in DNA and chromatin interactions.^50^ Importantly, prior studies lacked quantitative affinity measurements for isolated domains. Using biolayer interferometry (BLI), we determined the binding affinities of DBD1, DBD2, and BRCT for ssDNA, dsDNA, and G4 substrates under uniform conditions.

Our results revealed a refined hierarchy of DNA binding behavior. DBD1 displayed a clear preference for dsDNA, with 2:1 greater selectivity over that of ssDNA or G4. This suggests a specialized role for DBD1 in targeting BRCA1 to DSB where dsDNA predominates, potentially acting during the recognition of the blunt ends and the recruitment of nucleases for end resection in HR.^7–11^ In contrast, DBD2 bound all three substrates (dsDNA, ssDNA, and G4) with similarly high affinities, indicating that it functions as a general DNA-binding anchor. BRCT also bound all three structures, albeit with lower affinity (∼3–5 fold weaker than DBD2), suggesting that it may provide secondary contacts to scan the DNA for structural cues. DBD1 provides structure-selective recognition that may guide BRCA1 localization. DBD1’s strong binding to dsDNA could bring BRCA1 to DSB sites to initiate HR, where it cooperates with DBD2 and BRCT to stabilize dsDNA ends. However, when DBD1 binds to G4 DNA, it may tip the balance and redirect BRCA1 to the resolution of replication stress and telomere structures.^28,29^ Interestingly, the BRCT has been implicated also in chromatin remodeling and transcriptional regulation at G4-prone loci.^41,43^ Thus, domain cooperativity combined with modest differences in relative DNA binding affinity could facilitate the recruitment of BRCA1 to DSB or to noncanonical DNA structures such as chromatin and telomeres.

These interpretations are further supported by AlphaFold2 structural models. As shown in Figure 6, DBD1 and DBD2 are predicted to reside on opposite, solvent-accessible surfaces of BRCA1, suggesting different accessibility to DNA substrates. DBD1 is more prominently surface-exposed and well-positioned to engage dsDNA at DSB. DBD2 and BRCT, on the other hand, are localized on one face of the protein, where they may jointly function in DNA binding. BRCT’s relatively buried position within the full-length BRCA1 model may partially explain its lower affinity for DNA. While DBD1 and DBD2 are largely unstructured in solution, BRCT contains well-defined α-helical and β-sheet elements.^51^ We tested a possibility that secondary structure can form in DBD1 and DBD2 upon binding to DNA, but circular dichroism spectroscopy data (Supporting Information) showed that the two DBDs remain intrinsically disordered, suggesting that high affinity DNA binding is driven by their structural flexibility and adaptability. Nevertheless, full-length BRCA1 may undergo larger-scale rearrangements upon complex formation with DNA and other repair proteins, particularly in multiprotein assemblies that respond to damage or replication stress.

**Figure 6:**
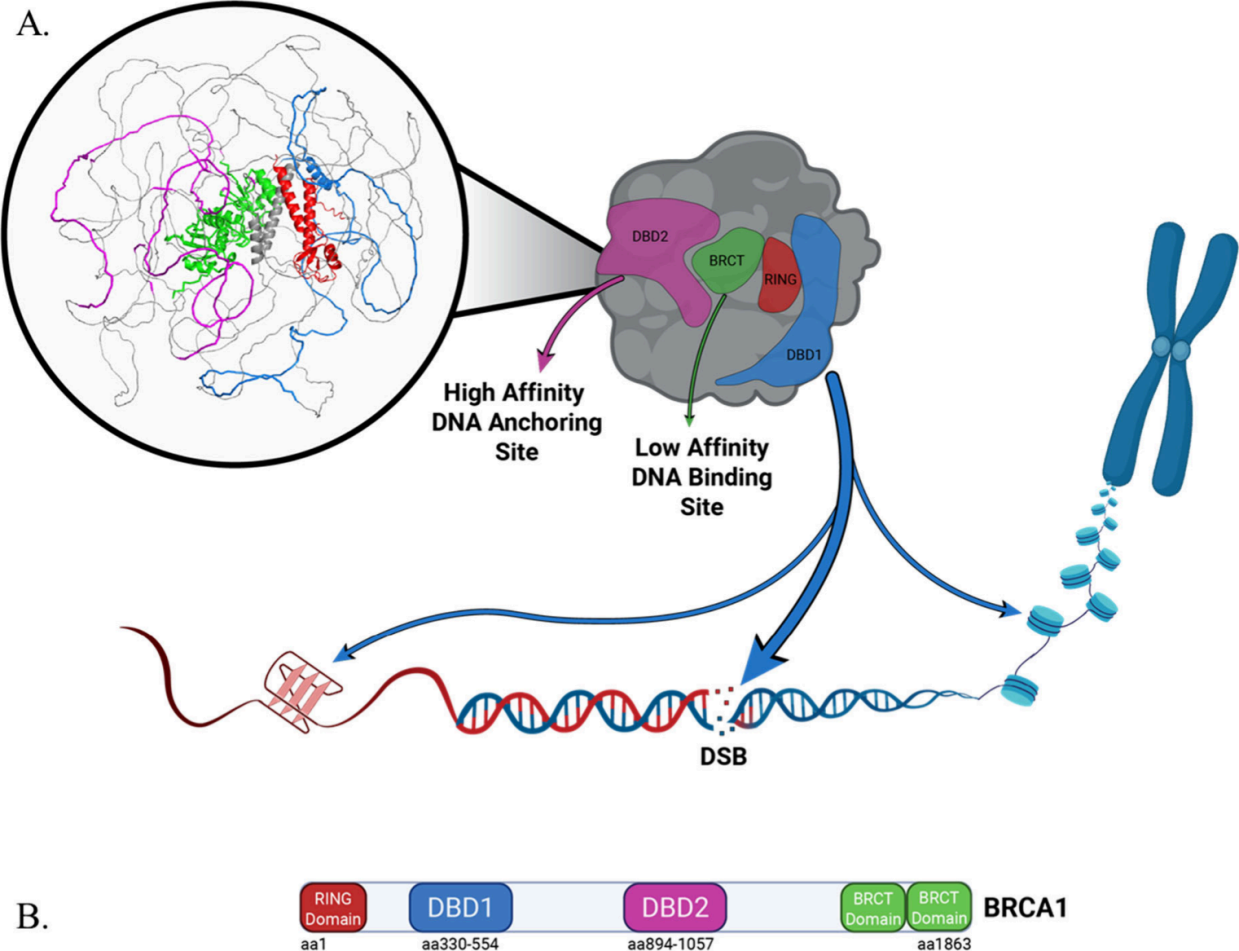
Model and domain maps of BRCA1. (A) Structural prediction of full-length BRCA1 using AlphaFold2 (right). Cartoon model (left) depicts the DNA binding properties of DBD1, DBD2, and the BRCT. (B) Cartoon map of BRCA1, highlighting domains RING (aa 1–109, red), DBD1 (aa 330–554, blue), and DBD2 (aa 894–1057, magenta), and the tandem BRCT repeats (aa 1646–1863, green). Cartoon was prepared using BioRender.

Our results confirm and add precision and resolution to previous findings. For example, Masuda et al.^25^ reported that residues 421–701 preferentially bind dsDNA over ssDNA, consistent with our observation that the DBD1 construct (aa 330–554), which overlaps this region, shows a distinct preference for dsDNA relative to ssDNA or G4. Similarly, the construct used by Sturdy et al.^46^ (aa 230–534) was reported to bind four-way functions, which may reflect DBD1 interacting with multiple dsDNA arms within branched substrates. This highlights the need for defined DNA substrates and DBD constructs in biophysical studies in order to make direct comparisons of DNA binding properties. Our findings diverge from earlier interpretations of DBD2 specificity. The larger fragment used by Brázda et al. (aa 444–1057) showed stronger binding to noncanonical structures such as G4 and cruciform DNA.^26^ Our DBD2 protein (aa 894–1057) retains this activity, but we find that it binds to dsDNA, ssDNA, and G4 with similarly high affinity, suggesting that DBD2 functions as the primary interaction site for multiple types of nucleic acid structures. We also observed that the BRCT domain binds to all three substrates but with weaker affinity compared to DBD2. This is consistent with prior studies by Yamane et al. and Matsumoto et al.,^42,52^ which demonstrates BRCT association with supercoiled and linear DNA. Our results further support the model proposed by Hu et al.,^41^ in which BRCT contributes to chromatin remodeling and transcriptional regulation. Given that our G4 substrate was derived from the human telomeric repeat sequence, BRCT’s ability to recognize this structure with moderate affinity may reflect its contribution to BRCA1 function in telomere surveillance.^53,54^

## Conclusions

Our findings provide quantitative evidence that the DNA binding activities of BRCA1 are modular. DBD1 shows a clear preference for dsDNA, while DBD2 exhibits strong, structure-independent binding to all tested substrates (dsDNA, ssDNA, and G4). The BRCT interacts with three substrates but with a lower affinity. These data support a model in which BRCA1 associates with DNA through coordinated contributions from its individual domains: DBD2 serves as a high-affinity anchor across diverse repair intermediates; BRCT provides auxiliary stabilization; and DBD1 confers structural discrimination. This enables BRCA1 to localize to DSB during HR via preferential binding to dsDNA or, alternatively, to telomeric or regulatory sites when DBD1 engages G4 DNA. This layered recognition mechanism may enable BRCA1 to adapt to the structural complexity of genomic DNA during replication, repair, and telomere maintenance. Future studies will be needed to determine whether a DBD1–DBD2 fusion (aa 330–1057) exhibits independent or cooperative binding and whether pathogenic mutations in these regions alter DNA affinity or specificity. Defining these DNA binding phenotypes may aid in understanding how BRCA1 variants contribute to genome instability and inform the interpretation of variants of uncertain significance in clinical studies.

## Supplementary Material



## Notes

Mass spectrometry and circular dichroism spectroscopy data (PDF)

## Notes

BRCA1, UniProt: P38398.

## ∇

Wayne State University, Detroit, MI 48202, USA

## §

University of Wisconsin-Madison, Madison, WI 53706, USA

## Abbreviations

*BRCA1*: breast cancer susceptibility gene 1
HR: homologous recombination
DSB: double-strand break
DBD: DNA binding domain
aa: amino acid
BRCT: BRCA1 C-terminal repeats
ssDNA or Bioss60R: single-stranded DNA
dsDNA or Biods60R-Cy3: blunt-end double-stranded DNA
G4 DNA or BioG4Telo: G-quadruplex DNA
53BP1: tumor suppressor p53-binding protein 1
PALB2: partner and localizer of BRCA2
BRCA2: breast cancer type 2 susceptibility protein
RAD51: DNA repair protein RAD51 homologue 1
NHEJ: nonhomologous end-joining
RING: really interesting new gene finger domain
FANCJ: Fanconi anemia complementation group J
BLI: biolayer interferometry
SA: streptavidin
