# Supplementary material for: Domain-Specific DNA Binding Activities of BRCA1 Reveal Substrate Preferences for Homologous Recombination and Telomere Regulation
Source: Biochemistry. 2025 Sep 4;64(18):3819–28. doi: 10.1021/acs.biochem.5c00333 (PMC12445000; doi:10.1021/acs.biochem.5c00333)
Supplement: Supplementary file 1 [file bi5c00333_si_001.pdf]

**SUPPORTING INFORMATION**

# Domain-specific DNA binding activities of BRCA1 reveal substrate preferences for homologous recombination and telomere regulation

*Kaitlin Lowran*[‡†], *Laura Campbell*[‡§], *Emma Cismas, and Colin G. Wu**

Department of Chemistry, Oakland University, Rochester, MI, 48309, USA

Present Addresses

† K.L.: Wayne State University, Detroit, MI, 48202, USA

§ L.C.: University of Wisconsin-Madison, Madison, WI, 53706, USA

*Email: colinwu@oakland.edu

‡ K.L. and L.C. contributed equally to this paper.

**Mass spectrometry (MS).** The DBD1 band was excised from gel, resuspended in water, and shipped to the Michigan State University Integrated MS Unit for analysis. The protein was digested with 500 ng rLys-C and 1 μg trypsin at 37 °C overnight. NanoLC-MS/MS separations were performed a Thermo Scientific™ Ultimate™ 3000 RSLCnano System. MS analysis was performed with a Q Exactive™ HF-X Hybrid Quadrupole-Orbitrap™ Mass Spectrometer. MS1 resolution was 60K at 200 m/z with a maximum injection time of 45 ms, AGC target of 3e6, and scan range of 300–1500 m/z. MS2 resolution was 30K at 200 m/z, with a maximum injection time of 54 ms, AGC target of 1e5, and isolation range of 1.3 m/z. HCD normalized collision energy was 28. Only ions with charge states from +2 to +6 were selected for fragmentation, and dynamic exclusion was set to 30 s. The electrospray voltage was 1.9 kV at a 2.0 mm tip to inlet distance.

The ion capillary temperature was 280°C and the RF level was 55.0. All other parameters were set as default. Protein identification was conducted by Proteome Discoverer™ Software version 2.5.0.400. Spectra were searched with Sequest against the reviewed Uniprot protein database (UP000000558), including DBD1, BRCA1 (Acc: P38398), contaminant sequences, trypsin (Acc:P00761), and LysC (Acc:Q02SZ7).

**Circular dichroism (CD) spectroscopy.** CD data was acquired using a JASCO J-815 spectropolarimeter (JASCO Inc.; Easton, MD, USA) equipped with a PTC-423S Peltier temperature control system. Protein and DNA samples were dialyzed into a buffer containing 20 mM Tris HCl (pH 7.5), 150 mM KCl, 1 mM DTT, and 5% glycerol. CD measurements were recorded at 25 °C across a wavelength range 200-260 nm for the BRCA1 DBD1 and DBD2. DNA substrates were titrated into the protein samples to assess changes in secondary structure upon binding. For each sample, five scans were collected and averaged. Reference spectra of buffer and DNA alone were subtracted from the averaged signal to obtain the final data.

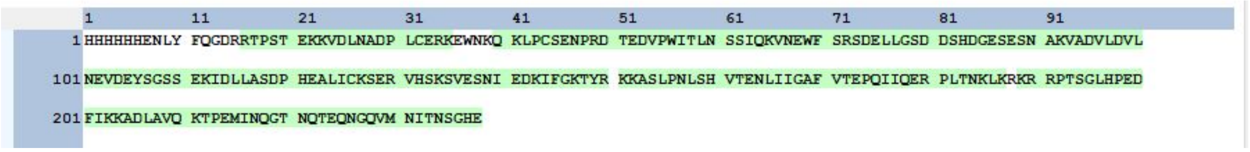


**Supporting Figure 1**. The amino acid sequence of recombinant DBD1 was confirmed by bottom-up tandem mass spectrometry. The expected DBD1 sequence is depicted with identified peptides highlighted in green. Sequence coverage was 92%. No peptides external to the DBD1 region were identified.

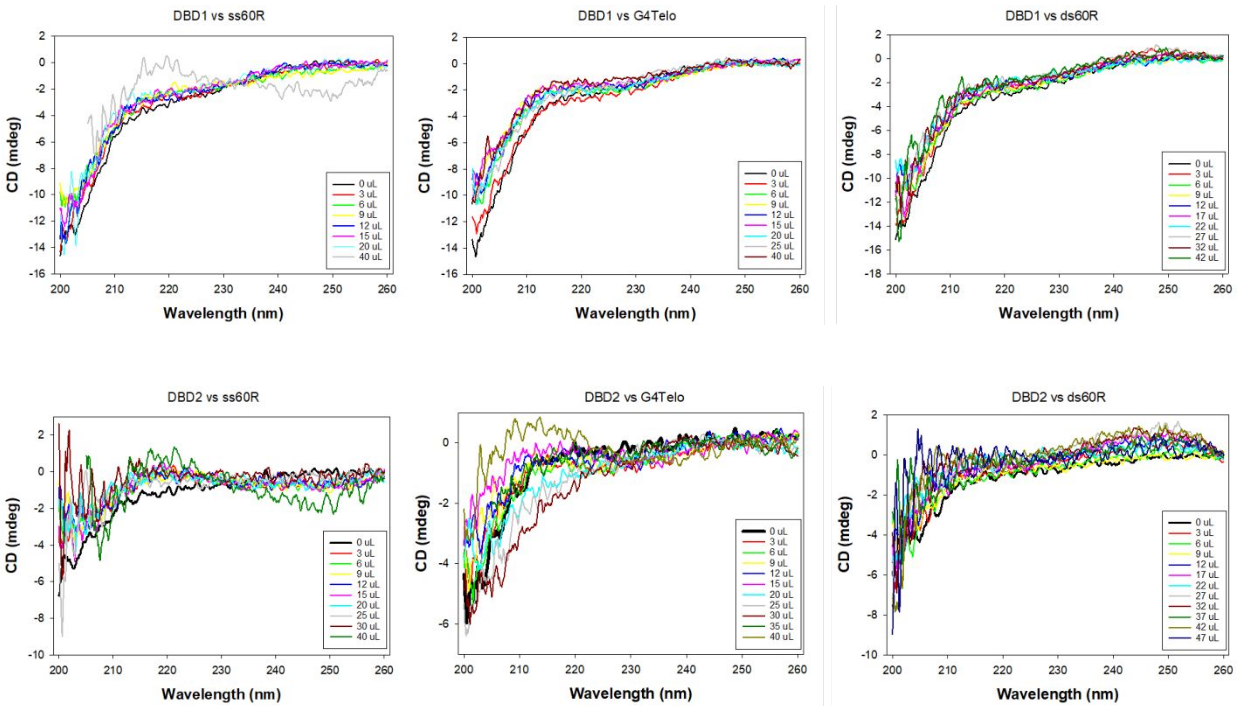


**Supporting Figure 2**. Circular dichroism spectra of DBD1 and DBD2 as a function of ssDNA, dsDNA, and G4 added. The results indicate that the domains remain intrinsically disordered upon binding to the DNA substrates.
